# Expression of TLR2/4 in the sperm‐storing oviduct of the Chinese soft‐shelled turtle *Pelodiscus sinensis* during hibernation season

**DOI:** 10.1002/ece3.1726

**Published:** 2015-09-23

**Authors:** Quanfu Li, Lisi Hu, Ping Yang, Qian Zhang, Yasir Waqas, Tengfei Liu, Linli Zhang, Shuai Wang, Wei Chen, Yuan Le, Shakeeb Ullah, Qiusheng Chen

**Affiliations:** ^1^ Laboratory of Animal Cell Biology and EmbryologyCollege of Veterinary MedicineNanjing Agricultural UniversityNanjingChina

**Keywords:** Hibernation, oviduct, soft‐shelled turtle, sperm storage, TLR2/4

## Abstract

The initiation of innate immunology system could play an important role in the aspect of protection for sperms long‐term storage when the sperms got into oviduct of turtles and come into contact with epithelium. The exploration of TLR2/4 distribution and expression in oviduct during hibernation could help make the storage mechanism understandable. The objective of this study was to examine the gene and protein expression profiles in Chinese soft‐shelled turtle during hibernation from November to April in the next year. The protein distribution of TLR2/4 was investigated in the magnum, isthmus, uterus, and vagina of the turtle oviduct using immunohistochemistry, and the gene expression of TLR2/4 was analyzed using quantitative real‐time PCR (qRT‐PCR). The results showed positive TLR2 protein expression primarily in the epithelium of the oviduct. TLR4 immunoreactivity was widely observed in almost every part of the oviduct, particularly in the epithelium and secretory gland membrane. Analysis of protein, mRNA expression revealed the decreased expression of TLR2/4 in the magnum compared with the isthmus, uterus, and vagina during hibernation. The protein and mRNA expression of TLR2 in the magnum, isthmus, uterus, and vagina was decreased in April compared with that in November. TLR4 protein and mRNA expression in the magnum, isthmus, uterus and vagina was decreased in November compared with that in April. These results indicated that TLR2/4 expression might protect the sperm from microbial infections. In contrast to the function of TLR2, which protects sperm during the early stages of hibernation, TLR4 might play a role in later stages of storage. The present study is the first to report the functions of TLR2/4 in reptiles.

## Introduction

Sperm storage, as a survival strategy beneficial for a variety of animals, including insects, fish, amphibians, reptiles, birds, and mammals, occurs in the female reproductive tract (Holt and Lloyd 2010; Holt 2011). To achieve fertilization, the sperm migrate within the female reproductive tract to encounter the oocytes (Sever et al. 2001). However, there is a discrepancy in the timing of ovulation in the female and the time of insemination by the male (Neubaum and Wolfner 1998; Suarez 2008b). To increase the chances of encountering sperm, the sperm are stored in the female genital tract (Birkhead and Møller 1993; Kuehnel and Kupfer 2012). In avian species, sperm storage tubules (SSTs) in the reproductive tract are beneficial to the survival of sperm (Long et al. 2003). In a previous study, we also revealed that the ultrastructural characteristics in the Chinese soft‐shelled turtle oviduct are closely associated with sperm storage (Xiangkun et al. 2008). A recent report showed that the origin of microvillus blebs (MvBs) on the apical tips of SST epithelial cells contributes to sustained sperm storage in the SSTs (Bakst and Bauchan 2015). Although there are many reports on both the occurrence and adaptive benefits of female sperm storage, few studies have been conducted to determine the mechanisms involved (Holt and Lloyd 2010). A study conducted in 2012 showed that the biogenic amines in Drosophila melanogaster females play essential roles in sperm storage (Avila et al. 2012). Recently, the study has been shown that a brain signaling pathway influences sperm storage (Lee et al. 2015).

There are many reports discussing the relationship between the innate immune system in the reproductive tract and the sperm (Palladino et al. 2008; Zandieh et al. 2015). The innate immune system is the major contributor to acute inflammation induced by microbial infection or tissue damage (Akira et al. 2006). Toll‐like receptors (TLRs) in the innate immune system play a key role in the modulation of immune and inflammatory responses in mammals against bacteria, viruses, and parasites (Belvin and Anderson 1996; Khan et al. 2013). TLR2/4 comprises two of the most well‐studied TLRs that detect invading pathogens and subsequently activate signal transduction cascades that result in the production of inflammatory cytokines and chemokines via effector cells and might also stimulate a peripheral immune response (Takeda and Akira 2004; Oliveira‐Nascimento et al. 2012). We hypothesized that these immunologic response products suppress the vitality of microorganisms that threaten the survival of resident sperm, generating an environment in which microorganisms exist together with sperm in the oviduct. Moreover, sperm can survive up to 2–15 weeks in domestic birds, including chickens, turkeys, quails, and ducks, depending on the species (Bakst et al. 1994; Bakst 2010), in contrast to the relatively short life span of mammalian spermatozoa (i.e., several days); however, unlike mammals and birds, in turtles, the male and female reproductive cycles are not synchronized (Licht 1984) and sperms are retained in the female oviduct for approximately 1 year (Liu et al. 2010).

It remains unknown whether the long‐term storage of the resident sperm in the turtle oviduct is associated with the innate immune system or whether there is some difference in the expression of TLR2/4 at different stages of storage, such as early (November) and late (April), during hibernation.

*Pelodiscus sinensis* (*P*. *sinensis*), commonly known as the Chinese soft‐shelled turtle, is a small freshwater animal, widely found throughout China. The *Pelodiscus sinensis* oviduct comprises the infundibulum, magnum, isthmus, uterus, and vagina. In this study, we focus on four parts of the oviduct: the magnum, isthmus, uterus, and vagina.

To our knowledge, there are few studies concerning the expression of TLRs in reptiles. Hence, the aim of this study was to determine TLR2/4 expression in the turtle oviduct containing sperm stored during different seasons (November and April). The protein expression of TLR2/4 was determined by immunohistochemistry, and TLR2/4 mRNA expression in the oviduct was analyzed by qRT‐PCR. We compared the expression of TLR2/4 in four different parts of the oviduct in November and April. Taken together, these data contribute to further studies on TLR2/4 expression in reptiles and provide a reference to explain the mechanism of sperm storage in reptiles.

## Materials and Methods

### Ethics statement

The Animal Ethics Committee at Nanjing Agricultural University reviewed the protocol and approved this study. The slaughter and sampling procedures were strictly performed according to the guidelines for the Ethical Treatment of Experimental Animals at Nanjing Agriculture University (Nanjing, China).

### Experimental soft‐shelled turtle and tissue collection

**Figure 1:**
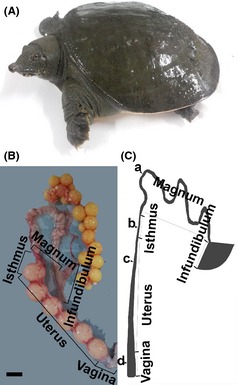
Photograph of soft‐shelled turtle and oviduct and its schematic representation. (A) Photograph of soft‐shelled turtle, *Pelodiscus sinensis*. (B) Gross anatomic view of soft‐shelled turtle oviduct showing the five main regions: infundibulum, magnum, isthmus, uterus, and vagina. (C) Schematic representation of the oviduct in *Pelodiscus sinensis*. The oviduct comprises the infundibulum, magnum, isthmus, uterus, and vagina. The present study focused on four points from which the materials were harvested: (a) magnum, (b) isthmus, (c) uterus, and (d) vagina. Scale bars: 1 cm (a and b).

Five sexually mature female *Pelodiscus sinensis* turtles (Fig. 1A), aged 3–4 years, of approximately the same size and weight were purchased from the Zhejiang Turtle Farm in the Zhejiang Province of China in April, and five female turtles were purchased in November. The living environment of the turtles was similar to the normal conditions in which the turtles live. The turtles were euthanized through the inhalation of 100% CO_2_, and the materials in four parts of the oviduct (Fig. 1B) (magnum, isthmus, uterus, and vagina) were subsequently harvested from the invariant sites (Fig. 1C). The collected samples were washed with PBS to remove resident sperm stored in the oviduct. Some samples were stored in paraformaldehyde and neutral formaldehyde, while other samples were snap‐frozen in liquid nitrogen. All protocols were approved through the Science and Technology Agency of Jiangsu Province (SYXK (SU) 2010‐0005).

### Light microscopy

The samples were fixed in neutral‐buffered formalin, embedded in paraffin, and serial sectioned (6 *μ*m) (Bian et al. 2013b). These sections were stained with hematoxylin and eosin (HE staining) reagent for observation using a light microscope. The slides were assessed by light microscopy using an Olympus BX53 microscope, and images were captured using a camera (Olympus DP73, Tokyo, Japan).

### Transmission electron microscopy (TEM)

The oviduct samples were cut into small blocks and fixed in a mixture of 2.5% glutaraldehyde in phosphate‐buffered saline (PBS; 4°C, pH 7.4, 0.1 mol/L) for 24 h. The blocks were postfixed in similarly buffered 1% osmium tetroxide for 1 h at room temperature after rinsing in PBS and washing with buffer. Ascending concentrations of ethyl alcohol were used to dehydrate the samples. Subsequently, the samples were infiltrated with a propylene oxide–Araldite mixture, and embedded in Araldite. The ultrathin sections (50 nm) were mounted on Formvar‐coated grids and stained with uranyl acetate and lead citrate for 20 min per step. The sections were examined and photographed using a transmission electron microscope H‐7650 (Bian et al. 2013a).

### Immunohistochemistry of TLR2/4 and microscopic analysis

The sections, after deparaffinization, were treated with 3% H_2_O_2_ at room temperature for 10 min to disrupt endogenous peroxidase. After three washes in the phosphate‐buffered saline (PBS), the slides were incubated in citrate buffer solution in boiling water for 15 min. After washing three times with PBS, the slides were incubated in the 5% BSA solution from the sABC Immunohistochemistry Kit (Boster, China) for 30 min in a humidified chamber to block nonspecific binding. The sections were incubated at 4°C for 15 h with 200 *μ*g/mL of a rabbit polyclonal antibody to human, mouse, and rat TLR4 (Boster, China). After washing five times with PBS, the sections were incubated with a goat anti‐rabbit IgG antibody (Boster, China) for 60 min. The sections were subsequently incubated for 30 min with sABC. Immunoreaction products were visualized after incubating the sections with 0.02% (w/v) 3–30 diaminobenzidine tetrahydrochloride (DAB) containing 0.001% (v/v) H_2_O_2_ in 0.05 mol/L Tris‐HCl (pH 7.6). The slides were rinsed with water and counterstained with hematoxylin. As a control, the first antibody was replaced with PBS. For TLR2, the staining was performed as discussed above using a rabbit polyclonal antibody to human, mouse, and rat TLR2.

Measurements and cell counts were performed using ImagePro 6.0 (Media Cybernetics, Silver Spring, MD). Positive results were expressed as the integral optical density (IOD). Samples were taken from five turtles, and three slides were prepared from each sample, and six areas per slide were selected to calculate the IOD.

### RNA extraction and qRT‐PCR

Total RNA was extracted from the oviduct samples using Total RNA Extraction Reagent (Beijing TransGen Biotech, Beijing, China). The concentration of the RNA was measured using BioPhotometer Plus (Eppendorf, German). Two micrograms of total RNA was reverse‐transcribed according to manufacturer's instructions in a 20‐*μ*L reaction containing 1 µL Random Primer, 10 µL 2× TS Reaction Mix, 1 µL RI Enzyme Mix, and RNase‐free water (Beijing TransGen Biotech). The reverse transcription protocol included 25°C for 10 min and 42°C for 30 min, followed by heat inactivation at 85°C for 5 min. The samples were stored at −20°C until further analysis. Gene‐specific primers were designed using Primer 3.0 software (Table 1) (Ye et al. 2012). Quantitative real‐time PCR was performed using the MyiQ2 Real‐Time PCR System (Bio‐Rad, California, USA). The PCRs were performed using SYBR^®^ Premix Ex Taq^™^ (Tli RNaseH Plus) (Takara‐bio, Dalian, China), according to the manufacturer's instructions. Each sample was analyzed in triplicate. The cycling protocol included 95°C for 1 min, followed by 40 cycles of 95°C for 15 sec and 61°C for 30 sec. The *β*‐actin gene was used as an internal control. The specificity of each PCR product was determined through melting curve analysis. The $2^{-\Delta \Delta C_{t}}$ method was used to analyze the PCR results (Livak and Schmittgen 2001), and the mRNA levels were expressed as fold‐changes relative to the mean value of first part of the oviduct (magnum).

**Table 1:** Oligonucleotide PCR primers

Gene	GenBank accession number	Primer sequence (5′–3′)	Orientation
TLR2	XM_006111559.1	CAGGCAGGGTGAAGGTATTGG	Forward
		CAGCCTGTGCAGCTAGAAAC	Reverse
TLR4	NM_001286933.1	GGACAGGAACGACACCTACG	Forward
		CACCCCTGGAATGAAGTCCC	Reverse
*β*‐actin	XM_006134862.1	AGACCCGACAGACTACCTCA	Forward
		CACCTGACCATCAGGCAACT	Reverse

### Statistical analysis

The results were expressed as the means means ± SE and analyzed using GraphPad Prism 5 software (San Diego, CA). The statistical analysis was performed using the Statistical Product and Services Solutions (SPSS, Chicago IL, USA) package, version 16.0. Student's *t*‐test and one‐way analysis of variance (ANOVA) were used to compare results between different groups. Differences were considered statistically significant when the *P* value was < 0.05.

## Results

### The microstructure and ultrastructure of the oviduct

**Figure 2:**
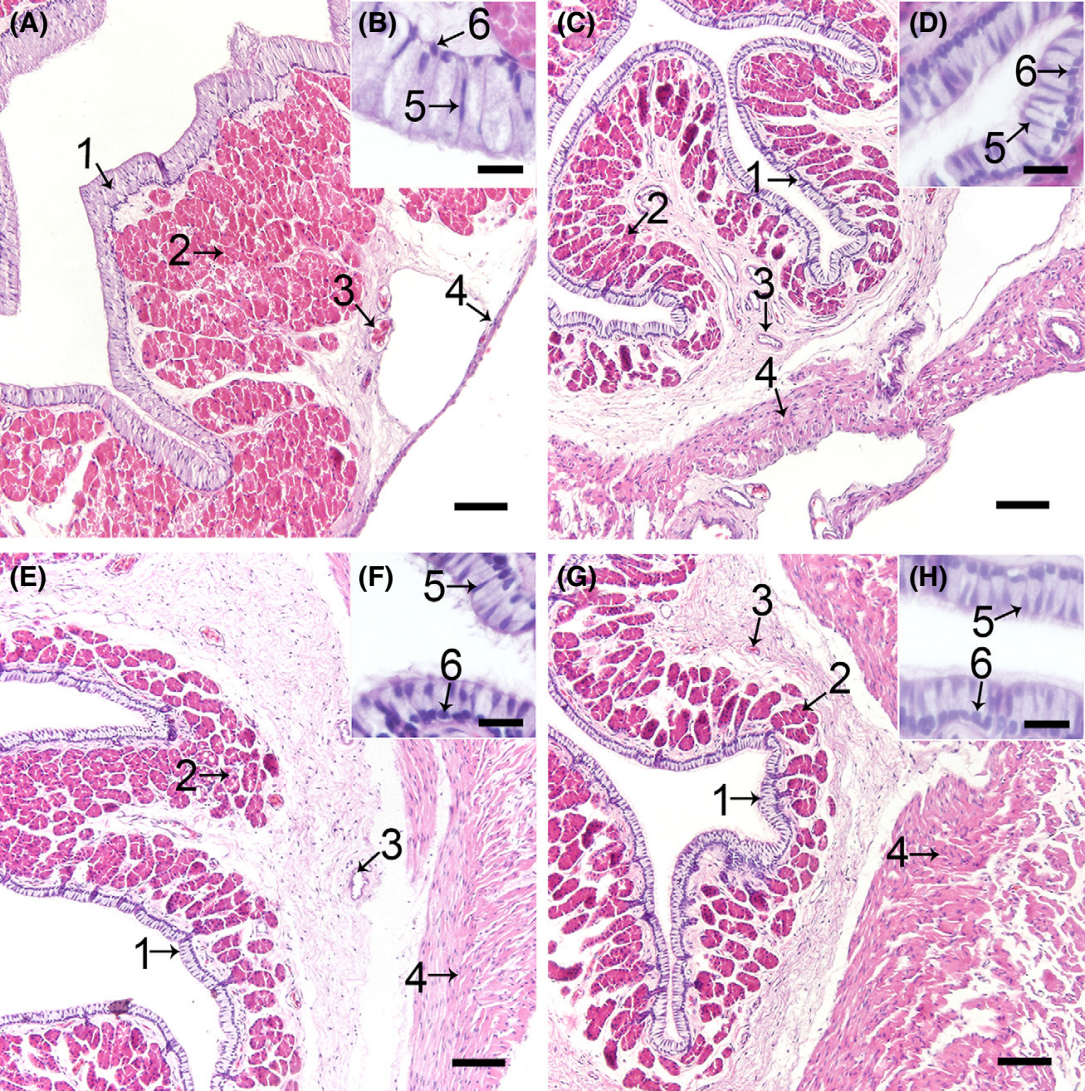
The Histological Structure of the Turtle Oviduct after H&E Staining. Cross‐sections of the magnum (A), isthmus (C), uterus (E), and vagina (G) of the oviduct, and the amplification of the epithelium in the magnum (B), isthmus (D), uterus (F), and vagina (H). Every part showed a similar structure. Epithelial cell layer (1), secretory gland (2) in the lamina propria, and blood vessels (3) in the submucous and muscle layers (4) are shown. In the epithelial cell layer, two cells types were observed: ciliated cell nucleus (5) and secretory cell nucleus (6). Scale bars: 100 *μ*m (A, D, E, and G) and 20 *μ*m (B, D, F, and H).

As shown in Figure 2, the oviduct comprises a mucous layer, submucous layer, muscle layer, and mantle layer. Ciliated and secretory cells were alternatively distributed. There was some discrepancy between the four different parts. In the magnum (Fig. 2A and B), the secretory cells were posited on the basilar membrane and the ciliated cells were posited among the secretory cells. The magnum primarily comprised secretory glands compared with the isthmus, uterus, and vagina. However, the isthmus (Fig. 2C and D), uterus (Fig. 2E and F), and vagina (Fig. 2G and H) primarily comprised muscle tissue.

**Figure 3:**
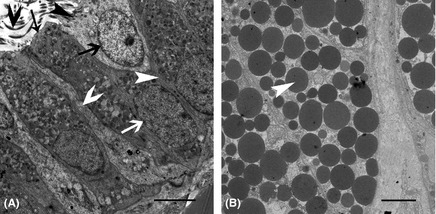
The Ultrastructure of the Epithelium and Secretory Glands. (A) Transverse sections throughout the epithelial cell; the ciliated cells have cilium (black fat arrow), which are located on the ciliated cell superior surface (black thin arrow), but not on the secretory cell superior surface (black thin arrow). The ciliated cell nucleus (black fat arrow) was similar in size to the secretory cell nucleus (white fat arrow). The junction (white thin arrow) between two secretory cells was observed. The junction (white fat arrow) between the secretory cell and the ciliated cell was also observed. We also observed the secretory cell basic membrane (white thin arrow). (B) Transverse sections throughout the secretory gland. The secretory glands were filled with many secretory particles (white thin arrow). Scale bar: 5 µm (A and B).

Conjoining cilium was observed on ciliated cells, establishing a continuous link between two cells (Fig. 3A). The secretory glands were filled with secretory vesicles (Fig. 3B).

### Immunolocalization of TLR2/4 in the oviduct in November and April

#### The expression of TLR2 protein in the oviduct in November was similar to that in April

**Figure 4:**
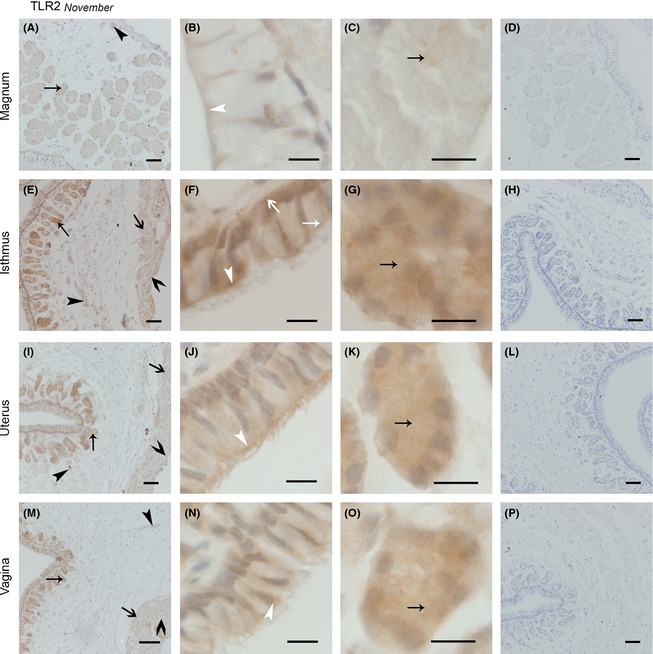
The Location of TLR2 Protein in November was Similar in the Magnum, Isthmus, Uterus, and Vagina. Cross‐sections of the magnum, isthmus, uterus, and vagina are depicted at a low magnification in A, E, I, and m, respectively; a higher magnification of the epithelium in the magnum, isthmus, uterus, and vagina is shown in B, F, J, and n, respectively; C, G, K, and o show the secretory glands in the magnum, isthmus, uterus, and vagina, respectively; and D, H, I, and p show the sections of the magnum, isthmus, uterus, and vagina incubated in PBS (negative control staining). No immunoreaction products were observed. All sections were counterstained with hematoxylin. Positive staining was observed on the ciliated cell superior part surface and cilium surface (white thin arrow head), secretory cell superior surface (white thin arrow), secretory cell basal membrane (white fat arrow), secretory glands vesicles membrane (black thin arrow), longitudinal muscle (black fat arrow), circular muscle (black fat arrow head), and blood vessel endothelium (black thin arrow head). Scale bars: 100 *μ*m (M), 50 *μ*m (A, D, E, H, I, L, and P), and 10 *μ*m (B, C, F, G, J, K, N, and O).

TLR2 protein expression in the oviduct in November is shown in Figure 4. In the magnum, slight TLR2 expression localized in the vascular endothelium (Fig. 4A), and strong expression localized to the superior part of the ciliated cell (Fig. 4B). Slight TLR2 expression was also detected on secretory vesicles membranes (Fig. 4C). We did not detect expression in the longitudinal or circular muscles (Fig. 4A). In contrast with the magnum, in the isthmus, TLR2 expression was observed in circular and longitudinal muscles and the vascular endothelium (Fig. 4E), the secretory cell surface, basal membrane, ciliated cell surface, cilium (Fig. 4F), secretory vesicles membranes (Fig. 4G). Expression in the uterus (Fig. 4I–K) showed no significant change compared with that in the vagina (Fig. 4M–O). However, the TLR2 expression was poor. Positive expression was observed in the surfaces of ciliated cells, the cilium, secretory vesicle membranes, longitude and circular muscles and the vascular endothelium.

**Figure 5:**
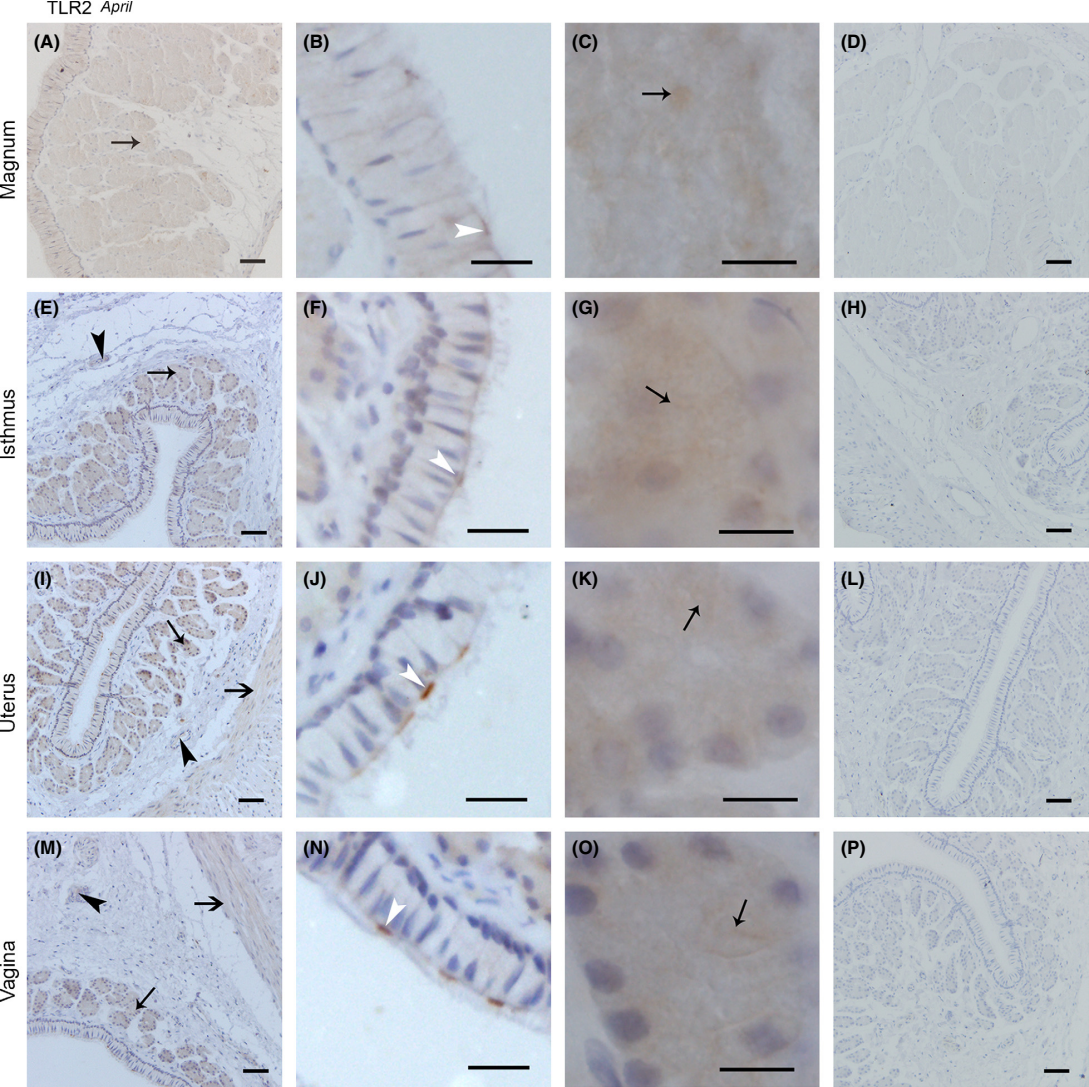
TLR2 protein Expression in April was Similar in the Magnum, Isthmus, Uterus, and Vagina. Sections of the magnum, isthmus, uterus, and vagina are shown at low magnification in A, E, I, and M, respectively; B, F, J, and N refer to the amplification of the epithelium in the magnum, isthmus, uterus, and vagina, respectively; the secretory glands in the magnum, isthmus, uterus, and vagina are described in C, G, K, and O, respectively; and D, H, I, and P represent the sections of the magnum, isthmus, uterus, and vagina incubated in PBS (negative control staining). No immunoreaction products were observed. All sections were counterstained with hematoxylin. Positive staining was observed on the ciliated cell superior part surface and cilium surface (white thin arrow head), secretory gland vesicles membrane (black thin arrow), longitudinal muscle (black fat arrow). Scale bars: 100 *μ*m (i), 50 *μ*m (A, D, E, H, L, M, and P), 20 *μ*m (B, F, J, and N), and 10 *μ*m (C, G, K, and O).

On the whole, the expression of TLR2 protein in April was different from that in November (Fig. 5). In the magnum, TLR2 expression was not observed in the muscles (Fig. 5A). The expression of TLR2 protein was observed in the superior surface of ciliated cells (Fig. 5B) and secretory gland vesicle membranes (Fig. 5C). The expression of TLR2 protein in the isthmus was more obvious than that in the magnum. With little expression in the circular muscle and vascular endothelium (Fig. 5E), increased expression was observed in the ciliated cell superior surface and the cilium (Fig. 5F). In the secretory glands (Fig. 5G), the expression of TLR2 protein in gland vesicle membranes was higher than that in the magnum. In the uterus (Fig. 5I–K) and vagina (Fig. 5M–O), the expression of TLR2 protein was similar to that in the isthmus, but poor expression was observed on the circular and longitude muscle in the uterus and vagina.

#### The expression of TLR4 protein showed a wider distribution than that of TLR2 protein in both November and April

**Figure 6:**
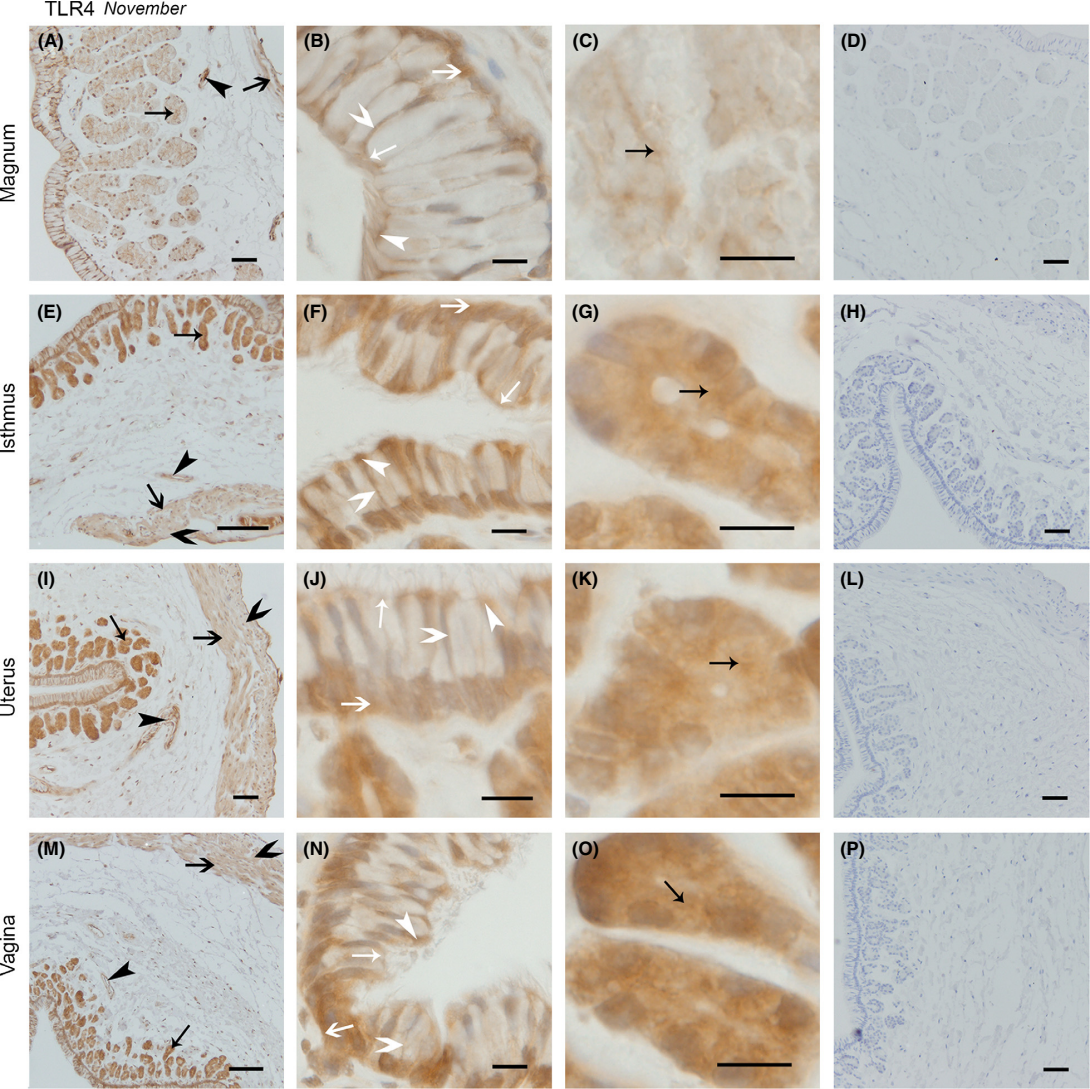
The Expression of TLR4 protein in November was Similar in the Magnum, Isthmus, Uterus, and Vagina. Cross‐sections of the magnum, isthmus, uterus, and vagina are shown at low magnification in A, E, I, and M, respectively; B, F, J, and n refer to the amplification of the epithelium in the magnum, isthmus, uterus, and vagina, respectively; the secretory glands in magnum, isthmus, uterus, and vagina are described in C, G, K, and o; respectively; and D, H, I, and P represent the negative controls for the magnum, isthmus, uterus, and vagina, respectively. No immunoreaction products were observed. All sections were counterstained with hematoxylin. Positive staining was observed on the ciliated cell superior surface and cilium surface (white thin arrow head), secretory cell superior surface (white thin arrow), secretory cell lateral membrane (white fat arrow head), secretory cell basal membrane (white fat arrow), secretory gland vesicles membrane (black thin arrow), longitudinal muscle (black fat arrow), circular muscle (black fat arrow head), blood vessel endothelium (black thin arrow head). Scale bars: 100 *μ*m (E and M), 50 *μ*m (A, D, H, I, L, and P), and 10 *μ*m (B, C, F, G, J, K, N, and O).

Generally, in the magnum, isthmus, uterus, and vagina, the expression of the TLR4 protein in November was widespread throughout the oviduct tissue, particularly in the secretory cell superior surface, basal membrane, ciliated cell superior surface, cilium, secretory gland, circular muscle, and vascular endothelium (Fig. 6). Some differences in the expression of TLR4 protein were observed along the oviduct from the magnum to the vagina. In the magnum, little expression was observed in the muscle (Fig. 6A), whereas increased expression was observed in the vascular endothelium (Fig. 6A). Positive expression was observed in the secretory cell superior surface, and little expression was observed in the basal membrane (Fig. 6B). Moreover, the widespread distribution of TLR4 expression was observed in the secretory gland vesicle membrane (Fig. 6C). In contrast with the magnum, positive expression was observed in the circular and longitude muscles and vascular endothelium in the isthmus (Fig. 6E). In secretory cells, the expression of TLR4 protein was observed in the superior surface, basal membrane, and lateral membrane, whereas in ciliated cells, the TLR4 protein expression was observed in the ciliated cell superior surface and cilium (Fig. 6F). On secretory glands (Fig. 6G), in different parts of the oviduct, increased TLR4 protein expression was observed in gland vesicle membranes than in the magnum. In contrast to the isthmus, similar TLR4 protein expression was observed in the uterus (Fig. 6I–K) and vagina (Fig. 6M–O).

**Figure 7:**
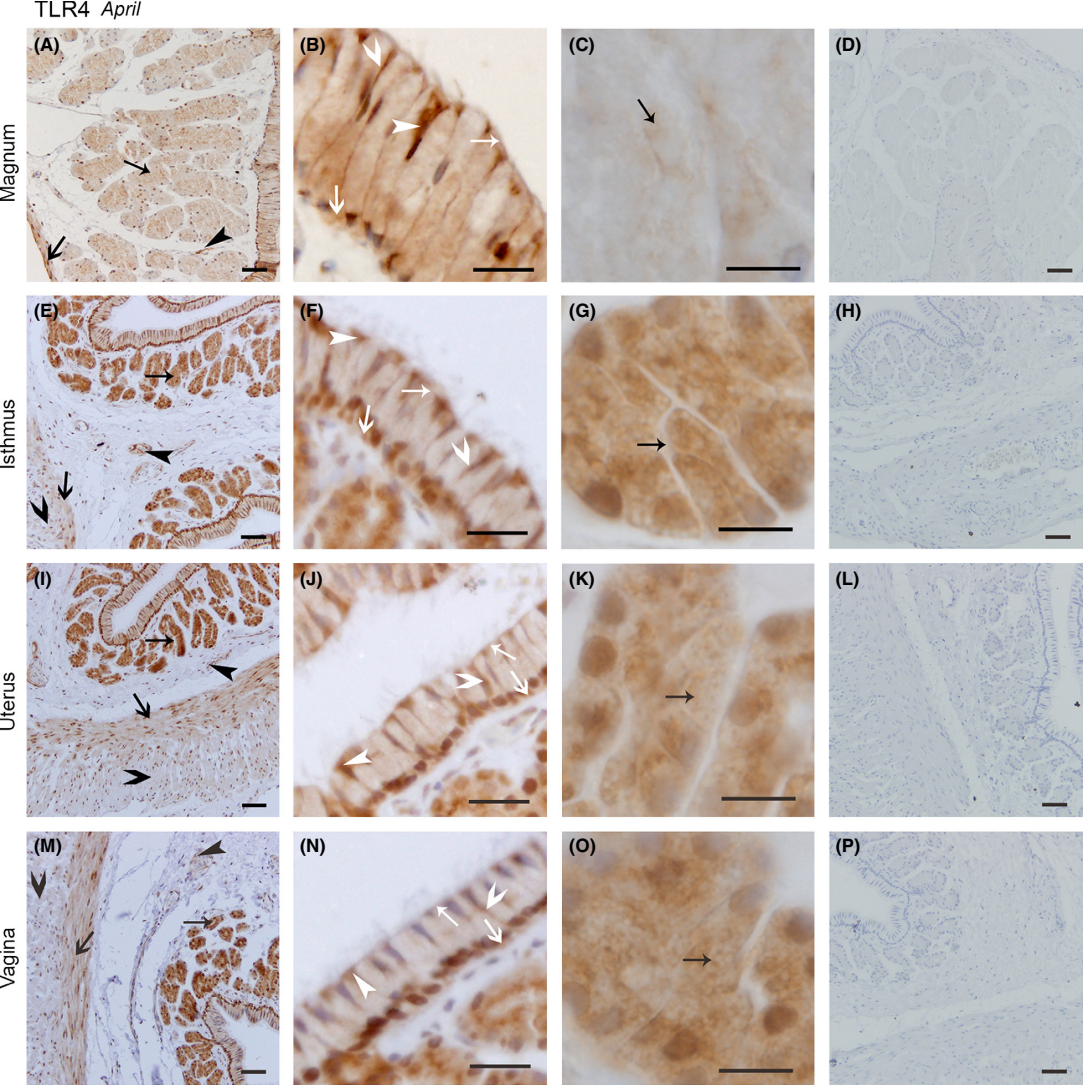
The Location of TLR4 protein was Similar in the Magnum, Isthmus, Uterus, and Vagina. Cross‐sections of the magnum, isthmus, uterus, and vagina are depicted in the pictures (A, E, I, and M) at low magnification: B, F, J, and n refer to the amplification for the epithelium in the magnum, isthmus, uterus, and vagina, respectively; the secretory glands in the magnum, isthmus, uterus, and vagina are described C, G, K, and O, respectively; and D, H, I, and p show the negative controls for the magnum, isthmus, uterus, and vagina, respectively. All sections were counterstained with hematoxylin. No immunoreaction products were observed. Positive staining was observed on the ciliated cell superior surface and cilium surface (white thin arrow head), secretory cell superior surface (white thin arrow), secretory cell lateral membrane (white fat arrow head), secretory cell basal membrane (white fat arrow), secretory gland vesicles membrane (black thin arrow), longitudinal muscle (black fat arrow), circular muscle (black fat arrow head), and blood vessel endothelium (black thin arrow head). Scale bars: 50 *μ*m (A, D, E, H, I, L, M, and P), 20 *μ*m (B, F, J, and N), and 10 *μ*m (C, G, K, and O).

The widespread distribution of TLR4 protein in the oviduct in April (Fig. 7) was also observed. In the magnum (Fig. 7A–C), the distribution of TLR4 protein did not change from that observed in November. Similarly, TLR4 expression in the isthmus (Fig. 7E–G), uterus (Fig. 7I–K), and vagina (Fig. 7M–O) was not different from that in November.

**Figure 8:**
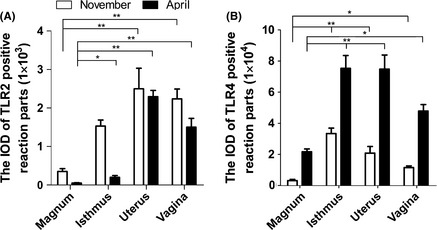
The IOD of TLR2/4 Positive Reaction Parts in Oviduct during Hibernation. (A) Image shows changes in numbers of TLR2‐positive reaction parts in four different parts of oviduct in two different months. (B) The IOD of TLR4‐positive reaction parts in oviduct during hibernation. Data are presented as mean ± SE of five turtles per group. Significant differences are identified as *, *P *<* *0.05; **, *P *<* *0.01.

During hibernation, for the TLR2/4 protein expression in oviduct, the results of the IOD were shown in Figure 8. The TLR2 protein expression, both in November (*F*_3,16_ = 11.152, *P *<* *0.001) and in April (*F*_3,16_ = 70.280, *P *<* *0.001), was significantly affected by distribution sites. Similarly, the protein expression of TLR4 was also significantly affected by distribution sites during hibernation (November: *F*_3,16_ = 22.939, *P *<* *0.001; April: *F*_3,16_ = 15.098, *P *<* *0.001).

#### Expression of TLR2/4 mRNA in the oviduct in April and November

**Figure 9:**
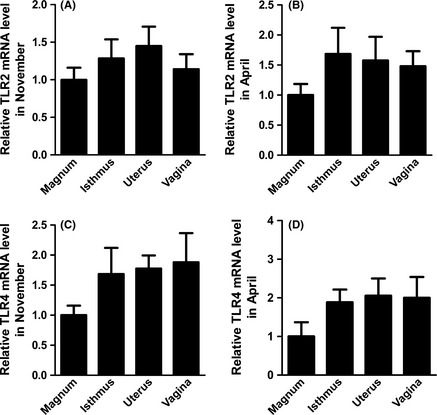
Validation of the Differential Expression of TLR2/4 mRNA Levels in Different Segments of the Turtle Oviduct using qRT‐PCR. The experimental procedure is described in the “Materials and methods.” The graphs show the mean ± SE of five turtles per group (*p*>0.05).

**Figure 10:**
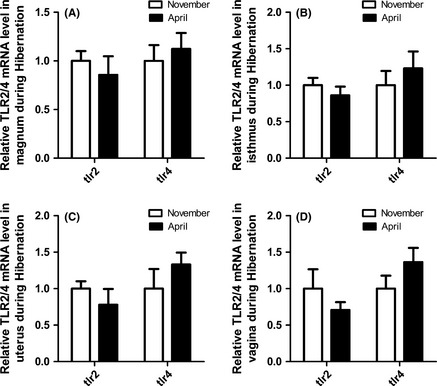
Comparison of the Differential Expression of TLR2/4 mRNA Levels at Different Storage Stages using qRT‐PCR. The experimental procedure is described in the “Materials and methods.” The graphs show the mean ± SE of five turtles per group(*p*>0.05).

On the whole (Fig. 9), both in April and November, TLR2/4 mRNA expression was lower in the magnum than in the isthmus, uterus, and vagina and the mRNA expression was not affected by distribution sites (TLR2, November: *F*_3,16_ = 0.767, *P = *0.529; TLR2, April: *F*_3,16_ = 1.607, *P = *0.227; TLR4, November: *F*_3,16_ = 1.560, *P = *0.238; TLR4, April: *F*_3,16_ = 1.435, *P *=* *0.270). As shown in Figure 10A–D, TLR2 mRNA expression in the magnum, isthmus, uterus, and vagina was higher in November than in April. However, the expression of TLR4 mRNA in the magnum, isthmus, uterus, and vagina was higher in April than that in November.

## Discussion

Previous studies have focused on the effects and functions of TLRs in the oviduct of mammals and birds (Zhan et al. 2013; Saeidi et al. 2014); however, no information has been reported concerning TLRs in the *Pelodiscus sinensis* oviduct. To our knowledge, this study is the first report demonstrating TLR2/4 protein and mRNA expression in the turtle reproductive tract and provides the first discussion of the changes in expression at different sperm storage stages. These findings demonstrated that TLR2/4 protein and mRNA is expressed in the oviduct of turtles, and the immunohistochemistry results showed that TLR2 protein was mainly localized to the membranes of ciliated cells, the cilium, and the membranes of secretory gland vesicles, while in other parts of the oviduct, less expression was observed. The expression of TLR4 protein, which was more widely distributed than TLR2 protein in the oviduct, was observed in nearly every part of the oviduct, particularly in the epithelial cell membrane and secretory gland vesicle membrane. At the protein and mRNA level, TLR2/4 expression was lower in the magnum than in the isthmus, uterus, and vagina during hibernation season. TLR2 protein and mRNA expression in the magnum, isthmus, and uterus decreased in April, compared with that in November. TLR4 protein and mRNA expression in the magnum, uterus, and vagina increased in April, compared with that in November.

Ozoe et al. (2009) suggested that the lower part of the tract expresses more TLR4 protein than the upper part in the hen oviduct. This is inconsistent with present study. In the our study, both in November and April, the expression of TLR2/4 mRNA and TLR2/4 protein in the turtle oviduct was higher in the lower part than in the upper part of the reproductive tract. When specific ligands bind to TLR2/4 receptors, the TLR2/4 signal pathway cascade is activated, leading to the release of a series of inflammatory factors, including IL‐1, IL‐6, IL‐10, IFN‐*β*/*α*, and TNF‐*β*, which protect the organism from damage (Takeuchi and Akira 2010; Vaure and Liu 2014). In addition, when sperm are inseminated into the oviduct, these cells pass through the vagina and reach the storage site, uterus and isthmus. In turtles, the sperm are primarily stored in the isthmus, with less in the uterus, little in the vagina, and none in the magnum (Liu et al. 2010). We proposed that TLR2/4 protein expression would increase at the site in which the sperm are stored. However, the TLR2/4 expression profile during the initiation of storage remains elusive (Peng et al. 2005). Based on the results obtained in the present study, TLR2 protein and mRNA expression was decreased in the magnum, isthmus, uterus, and vagina in April compared with that in November. Perhaps TLR2 protein plays a role in the early stages of hibernation during sperm storage. However, in the oviduct, TLR4 protein and mRNA showed greater expression in April than that in November, suggesting that TLR4 protein plays a role in last stages of hibernation. However, additional experiments are needed to determine the mechanism by which the sperm are protected.

Several reports have revealed that TLRs are expressed in various cell types, such as vascular endothelial cells, platelets, epithelial cells, adipocytes, skeletal muscle cells, pancreatic *β*‐cells, and hepatocytes (Akira and Takeda 2004). TLRs are also expressed in the cells of the central nervous system (CNS), including astrocytes, microglia, neurons, and cerebral vascular cells (Carty and Bowie 2011). Studies have shown that TLR2/4 expression is widely distributed throughout the reproductive tract (Young et al. 2004; Fazeli et al. 2005). Ozoe et al. (Ozoe et al. 2009) observed TLR4 immunoreactivity on the surface of the epithelium, and in spindle‐shaped subepithelial cells and leukocytes in the lamina propria of the isthmus, uterus, and vagina in the hen oviduct. This observation is consistent with the results obtained in the present study. However, in contrast to the findings reported herein, we also detected the expression of TLR2/4 protein in the epithelial cell membrane, cilium, muscles, vascular endothelium, and the membranes of secretory gland vesicles in the turtle oviduct.

Within the female reproductive tract, sperm proteins, membranes, and motility are modified to facilitate the fertilization of the egg upon release from the male (Suarez 2008a,b; Holt and Fazeli 2010). During this process, the sperm can receive protection from the oviduct. Studies have reported that large numbers of sperm remain aggregated in the lumen or embedded into the epithelium in this region, and the embedded sperm generally appeared normal in structure (Fritz and Turner 2002; Liu et al. 2010). However, most seminal components do not persist in mated females as long as the sperm are stored (Ram et al. 2005). Thus, it is likely that sperms migrate to specialized sites in which these cells are stored and got some materials from female (Wolfner 2011). Therefore, it is reasonable to imagine that the storage sites might provide a suitable environment to maintain the sperm (Heifetz and Rivlin 2010) and protect these cells from less supportive conditions elsewhere in the turtle reproductive tract. The secretory glands in turtles could provide the nutrition required for the consistent survival of sperm (Schnakenberg et al. 2011). Hence, the increased expression of TLR2/4 on the epithelium and secretory gland vesicle indicate that the sperm could be protected from invasion. However, the higher expression of TLR4 protein compared with TLR2 protein suggests that the protective effect of TLR4 protein would be more important than that of TLR2 protein. Because the interaction between resident sperm and TLR2/4 protein on epithelial cells separated from oviduct is now known, future in vitro studies are needed to identify mechanisms by which the sperm could escaped from microbial invasion.

In summary, the results of the present study demonstrated the distribution of TLR2/4 protein in the oviduct and indicated a role for TLR2/4 in the protection of the resident sperm in the oviduct against microorganisms. Moreover, these analyses suggest separate roles for TLR2 and TLR4 during the early and late stages of hibernation. Thus, these findings indicate a role for TLR2/4 in the protection of sperm during storage and contribute to further studies on TLR2/4 and the mechanism of sperm storage in reptiles.

## Conflict of Interest

None declared.

## Data Accessibility

All data are included in the manuscript and supporting information.
